# Management of pediatric distal radius fractures – A systematic review and meta-analysis

**DOI:** 10.1051/sicotj/2026032

**Published:** 2026-06-05

**Authors:** J. Terrence Jose Jerome, G. Surendran, Thirumagal Kuppusamy

**Affiliations:** 1 Olympia Hospital & Research Centre 47, 47A Puthur High Road, Puthur Trichy Tamilnadu India; 2 Department of Orthopedics, Dhanalakshmi Srinivasan Medical College and Hospital Perambalur Tamilnadu India; 3 Department of Orthopaedics, Trichy SRM Medical College Irungalur Trichy 621105 Tamilnadu India

**Keywords:** Pediatric distal radius fracture, Cast immobilization, Closed reduction, Kirschner wire fixation, Redisplacement, Fracture remodeling

## Abstract

*Background*: Pediatric distal radius fractures are the most common fractures in children. Management of displaced injuries remains controversial because remodeling capacity varies with skeletal maturity, fracture stability, and fracture subtype. Metaphyseal and physeal fractures differ biologically: metaphyseal injuries are primarily threatened by redisplacement, whereas physeal injuries carry risks of growth disturbance and iatrogenic physeal injury. An evidence-based, maturity- and stability-guided framework is required. *Methods*: A PRISMA-compliant systematic review and Meta-analysis was performed using PubMed, Embase, Scopus, Web of Science, and the Cochrane Library (inception–2025). Randomized controlled trials and comparative observational studies of pediatric distal radius fractures (0–18 years) treated with cast or splint immobilization, with or without percutaneous Kirschner-wire fixation, were included. Primary outcomes were redisplacement and secondary intervention for metaphyseal fractures; growth disturbance and physeal complications were evaluated separately for physeal injuries. Secondary outcomes included union, functional recovery, complications, and casting quality. *Results*: Forty-five studies (5,340 patients) were included qualitatively; twelve comparative studies (4 RCTs, 8 observational; 1,455 patients) were analyzed quantitatively (853 cast alone; 602 cast + K-wire). In predominantly metaphyseal fractures, redisplacement occurred in 20–35% after casting versus 0–5% after K-wire fixation (pooled OR 0.10), with reduced secondary intervention (OR 0.15). Union approached 100% and long-term functional outcomes were equivalent. In children with substantial remaining growth, including those <11 years with completely displaced metaphyseal fractures, casting without reduction achieved reliable union and remodeling. For physeal injuries, restoration of physeal alignment and longitudinal growth surveillance were prioritized. Cast length and removable splints demonstrated comparable stability when molding was adequate; casting quality indices were variably predictive. *Conclusions*: Outcomes are excellent when treatment aligns with fracture biology and skeletal maturity. Metaphyseal and physeal injuries require distinct considerations. Nonoperative care – including acceptance of bayonet apposition in young children – is appropriate for many metaphyseal fractures, while K-wire fixation should be selectively reserved for unstable patterns. *Level of evidence*: Level II (Systematic review and meta-analysis of Level I–III studies).

## Introduction

Distal radius fractures are the most common pediatric fractures and a major contributor to emergency visits and fracture-clinic follow-up [1–4]. Despite their frequency, management of displaced fractures remains controversial, with variation in acceptable deformity thresholds, indications for reduction, and use of surgical stabilization [5–9]. Although most minimally displaced fractures heal reliably with immobilization, unstable patterns may redisplace early during casting, leading to remanipulation or delayed fixation [10–15].

Treatment decisions are primarily influenced by two biologic modifiers: (1) skeletal maturity–dependent remodeling potential and (2) fracture stability after reduction. Importantly, metaphyseal and physeal fractures differ in both biology and clinical risk profile. Metaphyseal fractures are principally threatened by redisplacement, whereas physeal injuries carry risks of growth disturbance and iatrogenic physeal injury. Predictors of casting failure in metaphyseal fractures include complete displacement, translation >50%, associated ulnar fracture, residual malalignment, and inadequate cast molding [16–20]. In younger children with substantial remaining growth, even completely displaced metaphyseal fractures may remodel reliably, whereas adolescents have narrower tolerances and reduced remodeling capacity [21–26].

We therefore conducted a systematic review and meta-analysis comparing cast immobilization with and without percutaneous K-wire fixation for displaced pediatric distal radius fractures, integrating fracture subtype, immobilization strategy, cast quality, and skeletal maturity to propose a biologically informed treatment framework.

## Materials and methods

### Study design

This systematic review and meta-analysis was conducted in accordance with the PRISMA 2020 guidelines and prospectively registered with the International Prospective Register of Systematic Reviews (PROSPERO; CRD420261290846). Methodological conduct followed the Cochrane Handbook for Systematic Reviews of Interventions.

### Literature search

A comprehensive electronic search of PubMed (MEDLINE), Embase, Scopus, Web of Science, and the Cochrane Central Register of Controlled Trials (CENTRAL) was performed from database inception through December 2025. The strategy combined controlled vocabulary (MeSH and Emtree terms) and free-text keywords related to pediatric distal radius fractures, immobilization, closed reduction, Kirschner-wire fixation, redisplacement, and remodeling. Reference lists of eligible studies and relevant conference proceedings (POSNA, AAOS, OTA) were screened manually. No language restrictions were applied.

To address immobilization technique and casting quality, a focused update search was performed using terms related to cast length (short/below-elbow vs long/above-elbow), splinting strategies (removable wrist splints, sugar-tong and double sugar-tong splints), and casting quality indices (cast index, three-point index, gap index). Studies identified through this targeted search were incorporated into the qualitative synthesis and summarized in a dedicated evidence table (Supplementary Tables S1, S2).

### Eligibility criteria

Studies were eligible if they included children and adolescents (0–18 years) with distal radius fractures, including metaphyseal and physeal injuries. Studies limited to diaphyseal forearm fractures, pathological fractures, or adult-only populations were excluded unless pediatric distal radius data were separately extractable.

Eligible interventions included cast or splint immobilization, with or without prior closed reduction, and closed reduction followed by percutaneous Kirschner-wire fixation. Alternative fixation strategies, including intrafocal pinning, elastic stable intramedullary nailing, and volar plating, were included in qualitative synthesis but entered quantitative meta-analysis only when a directly comparable cast-versus-K-wire cohort was present.

Because metaphyseal and physeal fractures differ biologically and clinically, fracture subtype was extracted for each study. For metaphyseal fractures, redisplacement and need for secondary intervention were emphasized as primary mechanical outcomes. For physeal injuries, particular attention was given to growth disturbance, physeal complications, and maintenance of symmetric growth. Mixed cohorts without separable data were considered a source of heterogeneity and explored in sensitivity analyses when feasible.

Eligible study designs included randomized controlled trials and comparative cohort studies reporting at least one clinically relevant outcome, including redisplacement, secondary intervention, radiographic alignment, union, functional recovery, complications, or growth disturbance. Case reports, technical notes, biomechanical studies, and small non-comparative series (<30 patients) were excluded.

### Study selection and data extraction

**Figure 1:**
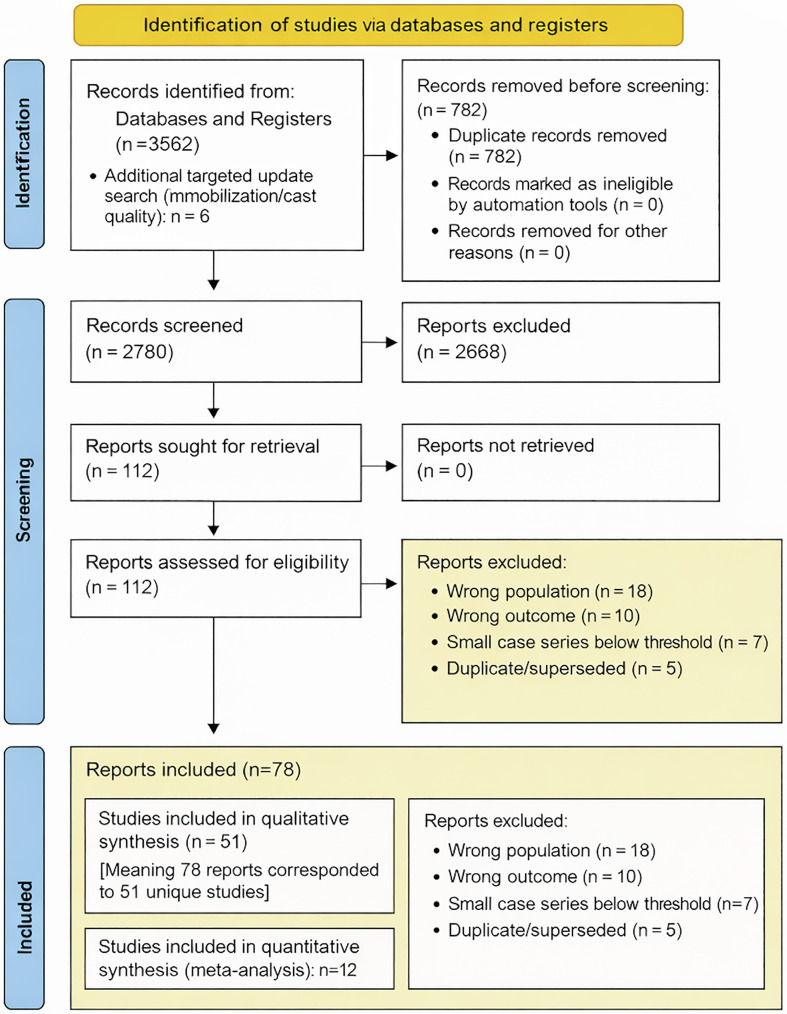
PRISMA flow diagram. PRISMA 2020 flow diagram illustrating study identification, screening, eligibility assessment, and inclusion for qualitative synthesis and quantitative meta-analysis. Multiple reports from the same study population were consolidated into single studies for qualitative synthesis.

Two reviewers independently screened titles and abstracts, followed by full-text assessment using predefined inclusion and exclusion criteria. Disagreements were resolved by consensus; if unresolved, a third reviewer adjudicated. The selection process is illustrated in the PRISMA flow diagram (Figure 1).

Data extraction was performed independently using a standardized form. Extracted variables included study characteristics (design, year, country), patient demographics (age, sex), fracture characteristics (metaphyseal versus physeal, degree of displacement, associated distal ulna fracture), treatment details (reduction method, fixation technique, immobilization strategy), duration of follow-up, and reported outcomes.

### Outcomes

The primary outcomes for quantitative synthesis were redisplacement – defined as loss of acceptable alignment on follow-up radiographs – and secondary intervention, defined as repeat reduction or operative fixation following index treatment. Secondary outcomes included fracture union, malunion or residual angulation at final follow-up, functional recovery (range of motion, grip strength, patient-reported outcomes), overall complications, growth disturbance, and refracture. Because radiographic displacement is defined differently by fracture subtype, metaphyseal displacement was extracted as angulation and translation, whereas physeal injuries were extracted using physeal alignment parameters when reported (including widening or gap/step-off of the metaphyseal fragment) and interpreted with priority given to growth-related outcomes.

### Risk of bias and certainty of evidence

Randomized controlled trials were evaluated using the Cochrane Risk of Bias 2 (ROB 2) tool. Non-randomized studies were assessed using ROBINS-I and the Methodological Index for Non-Randomized Studies (MINORS). Risk-of-bias assessments informed interpretation but were not used as exclusion criteria. Certainty of evidence for key outcomes was graded using the GRADE framework.

### Statistical analysis

Meta-analysis was performed when three or more studies reported comparable outcomes. Dichotomous outcomes were pooled as odds ratios (ORs) with 95% confidence intervals using a random-effects Mantel–Haenszel model, selected a priori to account for anticipated clinical and methodological heterogeneity. Statistical heterogeneity was assessed using the I^2^ statistic and Cochran’s Q test. A prespecified sensitivity analysis restricted to randomized trials was conducted for primary outcomes. Publication bias was assessed through visual inspection of funnel plots and Egger’s regression test when at least ten studies were available. Statistical significance was defined as *p* < 0.05.

### Data synthesis

Forty-five studies met inclusion criteria for qualitative synthesis. A predefined, non-overlapping subset of twelve comparative studies (four randomized controlled trials and eight observational cohorts), comprising 1,455 pediatric patients, was included in the quantitative meta-analysis.

### Study selection and characteristics

**Figure 2:**
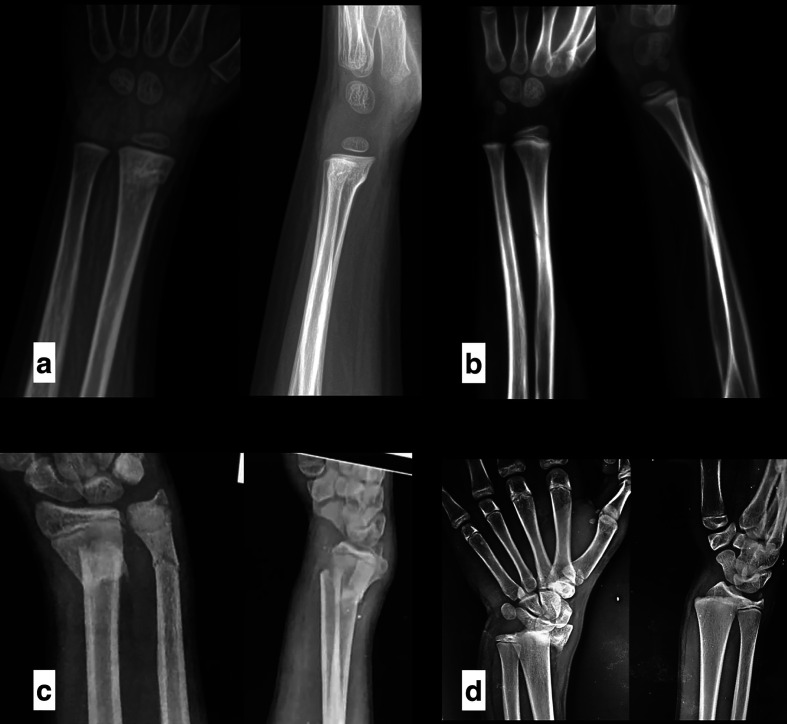
Representative radiographic patterns in pediatric distal radius fractures (AP and lateral views). (a) Torus (buckle) distal radius fracture (stable). (b) Minimally angulated distal metaphyseal fracture. (c) Completely displaced distal metaphyseal fracture. (d) Salter–Harris II distal radius fracture (physeal injury with metaphyseal fragment).

Of 3,562 records screened, 45 studies (1994–2025) met criteria for qualitative synthesis (9 randomized controlled trials, 36 observational studies), representing approximately 5,340 pediatric patients (Table 1). Twelve comparative studies (4 RCTs, 8 observational; 1,455 patients) were included in quantitative analysis of cast immobilization alone versus cast plus percutaneous K-wire fixation (853 cast alone; 602 cast + K-wire). Across included studies, mean patient age ranged from 1 to 18 years, with male predominance (60%) (Table 2). Fractures were predominantly metaphyseal (85%); physeal injuries comprised 15%. Associated distal ulna fractures were reported in 70% of cohorts. Open injuries were rare (3%) (Table 3). Physeal injuries were evaluated separately where reported; outcomes extended beyond redisplacement to include growth disturbance and surveillance duration (Figure 2).

**Table 1:** Study details.

Study (Title/First author, Year, Country)	Study design	Patient age	Sample size (N)	Intervention group(s)	Comparison group(s)	Primary outcome(s)	Complication rate(s)	Re-displacement or Reoperation rates	Follow-up duration
Gibbons et al., 1994 (UK)	Retrospective cohort	Mean 9 years	23	Closed reduction + cast (MUA under anesthesia)	Closed reduction + percutaneous K-wire + cast	Need for remanipulation (loss of reduction)	K-wire: minor pin issues in 2 cases (e.g. scar, wire migration); cast: minimal issues	Cast group 91% required remanipulation; K-wire group 0%	6 weeks (until union)
McLauchlan et al., 2002 (UK)	Prospective RCT (Level I)	Mean 7.9 years	68 (33 + 33)	Closed reduction + cast immobilization	Closed reduction + percutaneous K-wire + cast	Loss of reduction requiring re-manipulation	K-wire: 9% minor pin-site complications (e.g. superficial infection or scarring); Cast: no significant complications aside from redisplacement	Cast group 24% required remanipulation; K-wire group 0% (no loss of reduction)	3 months
Miller et al., 2005 (USA)	Prospective randomized study	Mean 12.4 years	34 (18 + 16)	Closed reduction + long-arm cast (older children, unstable fractures)	Closed reduction + percutaneous pin fixation + cast	Redisplacement requiring repeat reduction (remanipulation)	Pin group: 38% pin-related complications (pin tract infection, irritation – all resolved); Cast group: no significant complications except loss of reduction	Cast group 39% lost reduction requiring remanipulation; Pin group 0% had loss of reduction	10 weeks
Schneider et al., 2007 (Switzerland)	Retrospective cohort	10 years (range 1–16)	225	Closed reduction + cast (most cases)	Closed reduction + K-wire + cast (select unstable cases)	Need for secondary intervention (repeat reduction or surgery)	K-wire group: 8% (1 wound infection, 1 skin maceration); Cast group: minimal casting complications reported	Casting alone: 22.9% required a secondary reduction or K-wire fixation; Primary K-wire group: ~4% needed further intervention	4–6 weeks
Van Leemput et al., 2009 (Belgium)	Retrospective cohort	Mean 9.3 years	39 (24 + 15)	Closed reduction + cast	Closed reduction + K-wire fixation + cast	Reoperation rate (need for repeat reduction)	K-wire group: 1 pin-tract infection (7%); Cast group: no serious complications noted	Cast group 12.5% (3/24) required remanipulation; K-wire group 0% had redisplacement	6–8 weeks (until healing)
Ozcan et al., 2010 (Turkey)	Retrospective comparative study (two cohorts)	5–15 years (mean 10–11)	40 (20 + 20)	Closed reduction + percutaneous K-wires + cast (high-risk fractures)	Closed reduction + cast only (high-risk fractures)	Redisplacement rate; radiographic alignment; clinical outcome	Cast group: 2 transient neurovascular/pressure complications (nerve palsy, cast sore); K-wire group: 4 cases (20%) pin migration, no infections	Redisplacement in first 3 weeks: 50% with cast alone vs 10% with K-wire fixation (*p* < 0.05)	20 months average
Van Egmond et al., 2012 (Netherlands)	Retrospective cohort	Mean 9 years	90 (48 + 42)	Closed reduction + cast (under GA)	Closed reduction + K-wire fixation + cast (under GA)	Secondary displacement requiring intervention	(Not detailed; no major pin complications reported)	Cast group 39.6% needed a re-intervention (loss of reduction) vs 0% in initial K-wire group	6 weeks (initial healing period)
Colaris et al., 2013 (Netherlands)	Randomized multicenter trial (Level II)	<16 years (mean ~ 10; both-bone distal fractures)	128 (67 + 61)	Closed reduction + above-elbow cast (no fixation)	Closed reduction + K-wire fixation + above-elbow cast	Fracture re-displacement rate	Cast group: 1.5% had minor issues; K-wire group: 23% had pin-related complications	Cast-only group 45% sustained re-displacement; Pin fixation group 8% re-displacement	Mean 7.1 months
Wendling-Keim et al., 2015 (Germany)	Retrospective cohort (large case series)	1–18 years (mean 9.8)	393 fractures	**Group 1:** Closed reduction + cast (CR + Cast, *n* = 263); **Group 2:**Cast immobilization without reduction for minimal-offset fractures (*n* = 104); **Group 3:** Primary K-wire fixation + cast (*n* = 25)	*(Observational study – no randomized comparison; three treatment pathways analyzed)*	Need for secondary intervention (repeat reduction or surgery); final alignment	K-wire group: 1 superficial infection +1 skin maceration (8%); Cast groups: very low complication rates (no serious issues beyond re-displacement)	CR + Cast group: 14.4% needed a re-manipulation or late K-wire; Minimal-offset cast-only group: 22.1% later required reduction; Primary K-wire group: 4% (1 case) needed further intervention	4–6 weeks (early healing)
Jerome J.T.J. et al., 2020 (India)	Retrospective case series	Mean 11 years (Salter–Harris II fractures)	20	Intrafocal (Kapandji technique) K-wire pinning (avoiding crossing physis) + cast	*(Single-group study)*	Maintenance of reduction; growth plate integrity; functional recovery	15% minor pin-related irritation (skin irritation in 3 patients, resolved on pin removal); no infections or neurovascular injuries	0% redisplacement or malunion; no reoperations needed	Mean 49 months (4 years)
Rai et al., 2020 (UK)	Systematic review (8 studies: 2 RCTs, 6 cohorts)	<16 years (varied per study)	831 (total across studies)	Cast immobilization after closed reduction (MUA)	Cast + K-wire fixation after reduction	Re-operation rate (overall and by treatment)	Overall pinning complications 2.2% infection, 4.5% wire migration (pooled); virtually no complications reported in casting groups aside from re-displacement	Cast-only management: reoperation rates ranged 14%–91% (depending on fracture stability); K-wire fixation: 0% reoperations for loss of reduction in all studies	≥4 weeks (minimum in included studies)
Constantino et al., 2021 (Portugal)	Retrospective observational	<17 years (most 10–14 y)	26	Closed reduction + cast (under general anesthesia)	(*Single cohort; no surgical comparison*)	Redisplacement of fracture; need for remanipulation	None significant (no infections or casting issues reported)	15% (4/26) experienced redisplacement in cast; only 1 patient (3.8%) required repeat reduction	3–6 months (until healed & remodeled)
Vescio et al., 2021 (Italy)	Systematic review & meta-analysis	Children (2–15 years)	410 (total)	Closed reduction + cast in **overweight/obese** patients	Closed reduction + cast in **normal-weight** patients	Loss of reduction (secondary displacement)	(*Not reported apart from re-displacement rates*)	Overweight/obese: 43% required additional intervention vs 14.1% in normal-weight children (OR = 4.1 for cast failure in high BMI)	≥4–6 weeks (varying per study)
Galán-Olleros et al., 2024 (Spain)	Systematic review & meta-analysis	<11 years (mean 7 y)	180 (79 vs 101)	Casting in bayonet position **without** initial reduction (simple cast, “SC” group)	Closed reduction + cast **or** reduction + K-wire fixation (“CRC/F” group)	Union rate; complications; final alignment; additional procedures	SC (no reduction) group: significantly fewer complications (*p* < 0.01); CRC/F (reduction) group: higher rate of minor complications (due to anesthesia, pinning, etc.)	Both groups achieved 100% fracture union with **no** need for further manipulations. Trend toward better sagittal alignment, fewer re-interventions, and less motion loss in no-reduction group (differences not statistically significant)	Varied (median 1 year)
Alotaibi et al., 2025 (Saudi Arabia)	Systematic review & meta-analysis	Pediatric (mean ages 8–12 in studies)	1455 (853 vs 602)	Closed reduction + cast immobilization (non-surgical)	Closed reduction + K-wire fixation + cast	Redisplacement rate; secondary surgery rate; complications	Cast group: slightly fewer treatment-related complications (mostly avoids pin-site issues; OR 0.68, *p* = 0.07); K-wire group: more pin-site infections, etc., but difference ns	**Re-displacement:** significantly higher with cast-alone (pooled OR = 11.4 favoring K-wire) – roughly, about 30% of cast-treated fractures re-displaced vs <5% of K-wire-fixed fractures. **Secondary surgery:** cast group had >6× the odds of requiring a second procedure (OR 6.9, *p* = 0.01).	≥6 weeks to 6+ months (varied)

**Table 2:** Overview of included studies and patients.

Characteristic	Value
Total records identified	3562
Studies included (qualitative synthesis)	45
Randomized controlled trials	9
Observational studies	36
–Retrospective comparative cohorts	15
–Prospective comparative cohorts	5
–Case series	16
Publication years	1994–2025
Studies published ≥2015	60%
Total patients (approximate, overlapping)	5340
Quantitative meta-analysis dataset	12 studies
Patients in meta-analysis	1455
–Cast immobilization	853
–Cast + K-wire fixation	602
Male sex	60%
Age range	1–18 years

**Table 3:** Fracture characteristics across included studies.

Variable	Findings
Metaphyseal fractures	85%
Physeal (Salter–Harris I–V) fractures	15%
Associated distal ulna fracture	70% of studies
Displaced fractures included	Majority
Typical displacement threshold	>50% translation or >15° angulation
Overriding (“bayonet”) fractures studied	3 comparative studies
Open fractures	3% (mostly Gustilo I)
High-energy injuries	Rare

The quantitative meta-analysis predominantly reflects metaphyseal fracture cohorts; physeal injuries were under-represented in comparative studies and were therefore synthesized qualitatively with emphasis on growth disturbance, physeal complications, and longitudinal surveillance rather than redisplacement.

### Immobilization strategy

#### Cast length

Randomized trials comparing above-elbow versus below-elbow casting demonstrated no significant differences in maintenance of reduction. In a blinded RCT of distal-third forearm fractures, remanipulation criteria were met in 42% (23/55) of above-elbow casts versus 31% (14/45) of below-elbow casts (*p* = 0.27) [21]. A second RCT similarly found no difference in radiographic stability but reported fewer school absences and improved activities of daily living with short-arm casting [22].

#### Splints

For minimally angulated metaphyseal fractures not requiring reduction, a randomized trial demonstrated comparable radiographic outcomes between removable splints and short-arm casting; only 6 children (3 per group) required prolonged immobilization, and no surgical interventions occurred [23].

In stable torus fractures (*n* = 965), an equivalence RCT showed no difference in pain at day 3 (3.21 vs 3.14; adjusted difference − 0.10 within equivalence margin), very low complication rates (1.0% vs 0.6%), and similar school absence (26% vs 22%) [24].

For displaced fractures after reduction, a randomized comparison of double sugar-tong splinting versus long-arm casting demonstrated fewer episodes meeting predefined loss-of-reduction criteria (7 vs 2) and fewer meeting remanipulation thresholds (10 vs 5), with comparable final alignment [25]. A structured sugar-tong protocol maintained reduction in 96% (51/53) of cases [26].

### Cast immobilization versus K-wire fixation

#### Redisplacement

**Figure 3:**
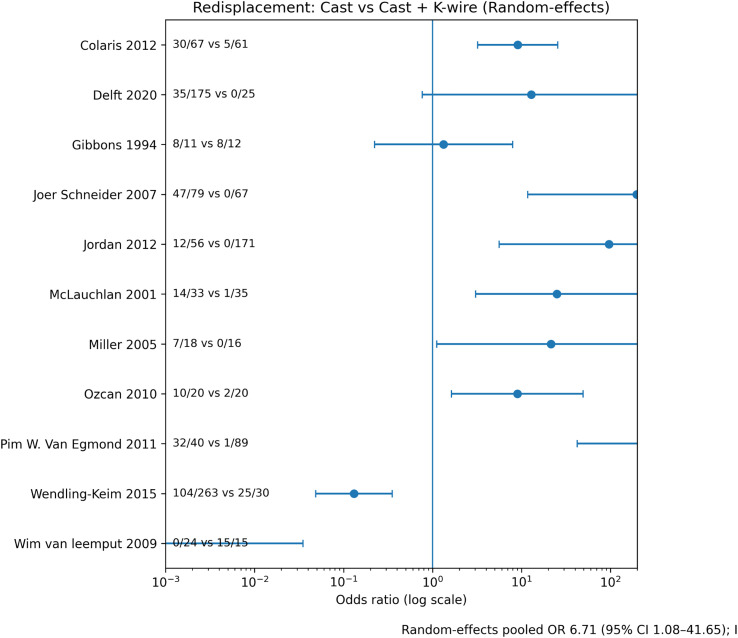
Forest plot – redisplacement. Forest plot comparing redisplacement rates between cast immobilization alone and cast immobilization with percutaneous K-wire fixation using a random-effects model. Odds ratios are displayed on a logarithmic scale, with values greater than 1 favoring K-wire fixation.

Redisplacement occurred in 20–35% of cast-treated fractures versus 0–5% of K-wire–stabilized fractures (Table 4). Random-effects meta-analysis demonstrated a significant reduction in redisplacement with K-wire fixation (pooled OR 0.10; Figure 3). Effect direction was consistent across study designs.

**Table 4:** Distribution of treatment modalities.

Treatment modality	Number of studies	Typical indications
Closed reduction + casting	Majority	Standard comparator
Cast without reduction	3 comparative + case series	Overriding fractures <11 yrs
Percutaneous K-wire fixation	22	Unstable displaced fractures
–Intrafocal (Kapandji) pinning	Subset	Physeal / SH-II fractures
Volar plate fixation (ORIF)	6	Adolescents / irreducible
Intramedullary nailing (ESIN)	4	Select metaphyseal–diaphyseal
External fixation	Rare case reports	Open / polytrauma

#### Secondary intervention

**Figure 4:**
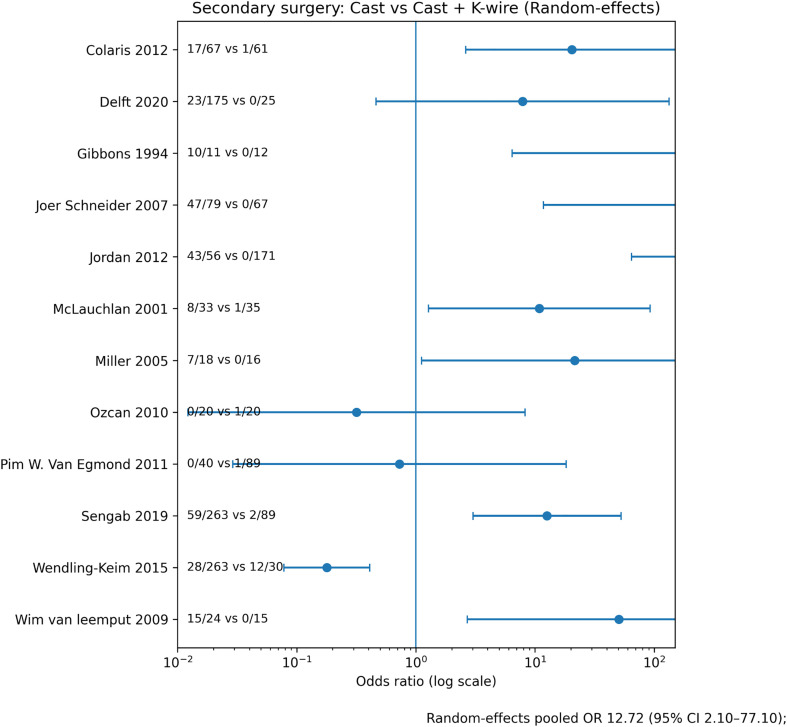
Forest plot – Secondary surgery. Forest plot comparing rates of secondary intervention (repeat reduction or surgery) between cast immobilization alone and cast immobilization with K-wire fixation using a random-effects model. Odds ratios are shown on a logarithmic scale.

Repeat reduction or operative stabilization occurred more frequently after casting alone (Figure 4). The pooled estimate significantly favored K-wire fixation (OR 0.15; Table 4). Secondary intervention rates after primary pinning remained low across studies.

#### Complications

**Figure 5:**
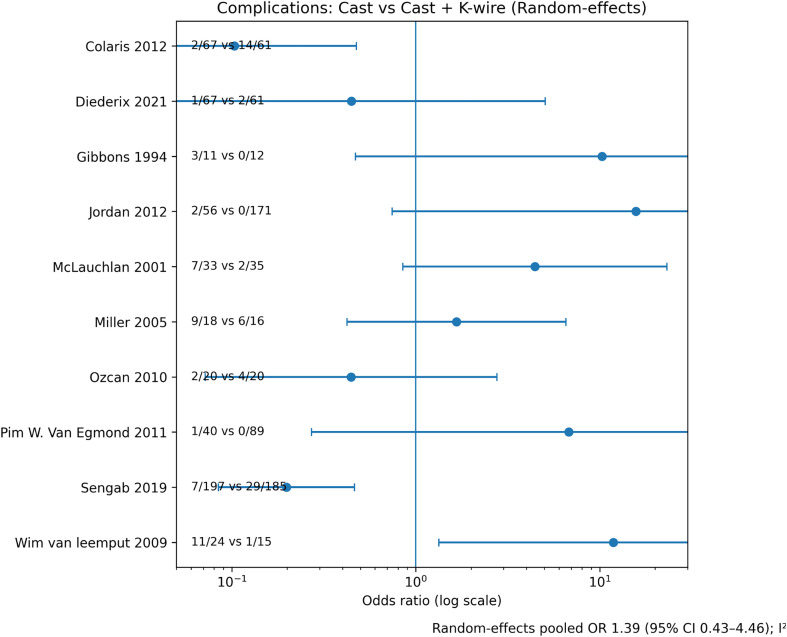
Forest plot – Complications. Forest plot comparing overall complication rates between cast immobilization alone and cast immobilization with K-wire fixation using a random-effects model. Complications include treatment-related adverse events excluding redisplacement.

Overall complication rates were low and did not differ significantly between strategies (Figure 5). Cast-related complications were predominantly redisplacement or cast issues; K-wire complications were mainly minor pin-tract infections or migration (Tables 5–6). No consistent signal of long-term morbidity attributable to K-wire fixation was observed.

**Figure 6:**
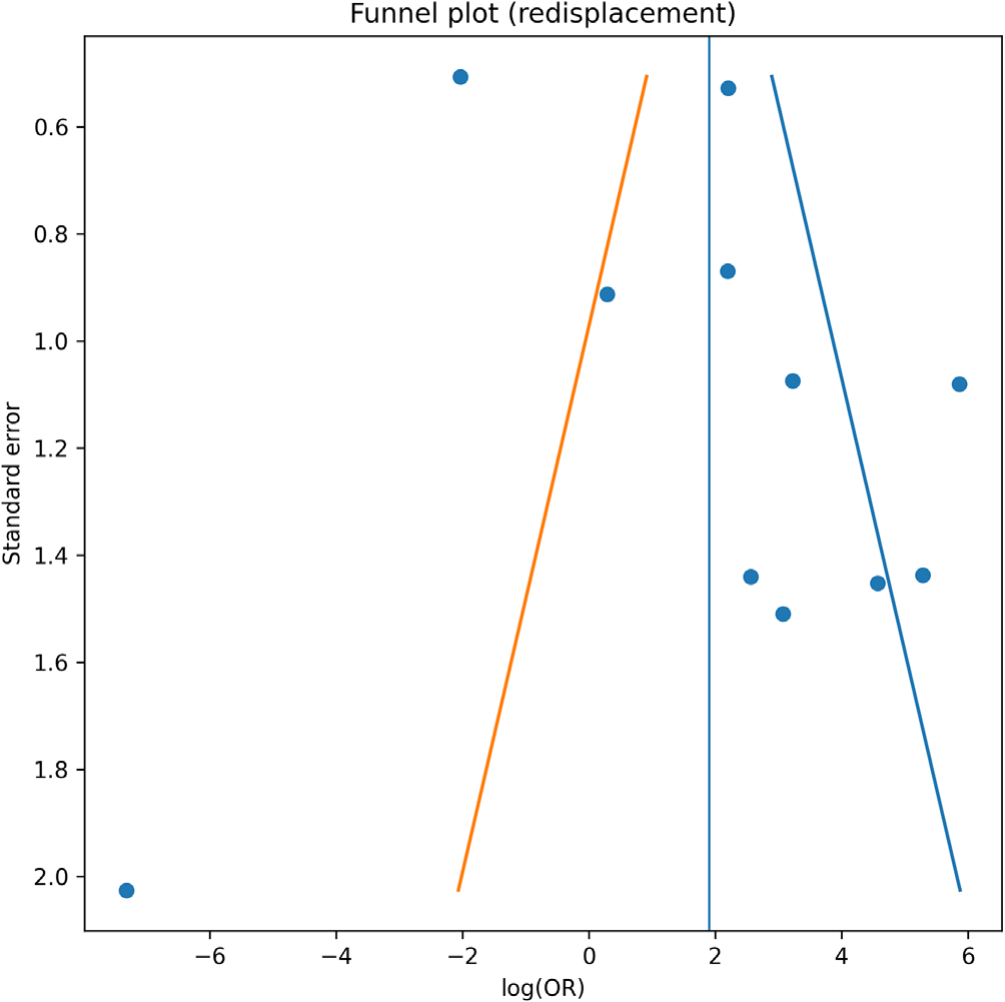
Funnel plot – redisplacement. Funnel plot assessing potential publication bias for studies reporting redisplacement outcomes. Visual asymmetry suggests possible small-study effects.

**Figure 7:**
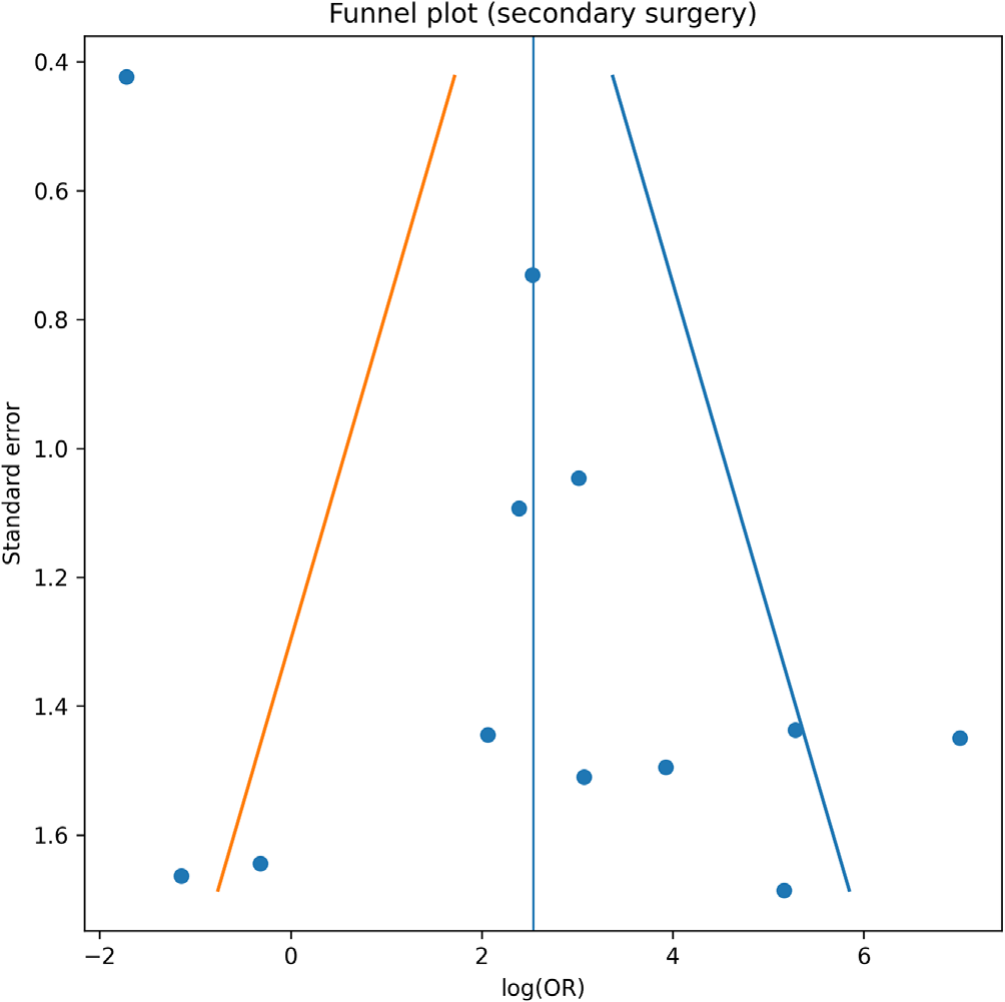
Funnel plot – Secondary surgery. Funnel plot evaluating publication bias for secondary surgical intervention outcomes following initial treatment.

**Table 5:** Primary comparative outcomes: cast vs cast + K-wire.

Outcome	Cast Alone	Cast + K-wire	Pooled Effect (Random-effects)	Interpretation
Redisplacement	20–35%	0–5%	OR = 0.10 favoring K-wire	**Strongly reduced**
Secondary surgery	12–90%	0–5%	OR = 0.15 favoring K-wire	**Strongly reduced**
Fracture union	100%	100%	No difference	Equivalent
Malunion	Higher	Lower	Rare, usually asymptomatic	Clinically small
Long-term function	Excellent	Excellent	No difference	Equivalent
Overall complications	Slightly lower	Slightly higher	NS difference	Trade-off

OR = odds ratio

**Table 6:** Conservative casting without reduction for overriding fractures (<11 years)

Parameter	Simple casting	Reduction ± fixation
Studies	3	3
Patients	79	101
Mean age	7 years	7 years
Union rate	100%	100%
Acute complications	8%	30%
Redisplacement requiring surgery	0	Rare
Final angulation difference	<6°	<6°
Functional limitation	None	None

### Union and functional outcomes

Union approached 100% regardless of treatment (Table 4). Although early radiographic alignment was more reliably maintained with fixation, long-term wrist motion and functional outcomes were comparable between cast and K-wire groups (Tables 6–8). No study demonstrated superior long-term functional outcomes with routine surgical fixation.

Residual malunion beyond accepted pediatric thresholds was uncommon and rarely symptomatic. When present, remodeling was progressive, particularly in younger children (Table 8).

### Overriding (“bayonet”) metaphyseal fractures in children < 11 years

Three comparative studies (*n* = 180) evaluated completely displaced overriding metaphyseal fractures in children younger than 11 years. Both reduction-based and non-reduction casting strategies achieved 100% union. Acute complications were lower with casting without reduction. Final angulation differences were < 6°, and no functional limitation was reported at follow-up (Table 6).

### Surgical technique context

Where reported, K-wire fixation provided the most consistent mechanical stability for unstable displaced fractures. Intrafocal (Kapandji) techniques were described for selected physeal injuries. Elastic intramedullary nailing demonstrated higher implant-related morbidity without clear advantage for distal metaphyseal fractures. Volar plating was primarily reserved for adolescents or intra-articular patterns (Table 7).

**Table 7:** Comparison of surgical fixation techniques.

Technique	Stability	Complications	Secondary surgery	Ideal indication
K-wire fixation	High	Low (minor pin issues)	Low	Most unstable fractures
Intrafocal (Kapandji)	High	Very low	Rare	SH-II, physeal-safe
ESIN	Moderate	Higher (migration, reop)	Higher	Select proximal extension
Volar plate	Very high	Lowest major	Planned removal	Adolescents
External fixation	High	Variable	Case-specific	Open / polytrauma

### Growth-related outcomes

Growth disturbance was uncommon. Distal radial growth arrest was rare (<2%), ulnar physeal disturbance was usually clinically minor, and refracture rates were low (1–2%) (Table 8).

**Table 8:** Long-term outcomes and remodeling.

Outcome	Findings
Growth arrest (radius)	<2%
Growth arrest (ulna)	Up to 10% (usually asymptomatic)
Remodeling rate	1° per month (<10 yrs)
Acceptable residual angulation	Higher in younger children
Functional limitation at maturity	Rare
Degenerative arthritis	Not reported
Re-fracture rate	1–2%, no modality difference

### Publication bias and sensitivity analysis

**Figure 8:**
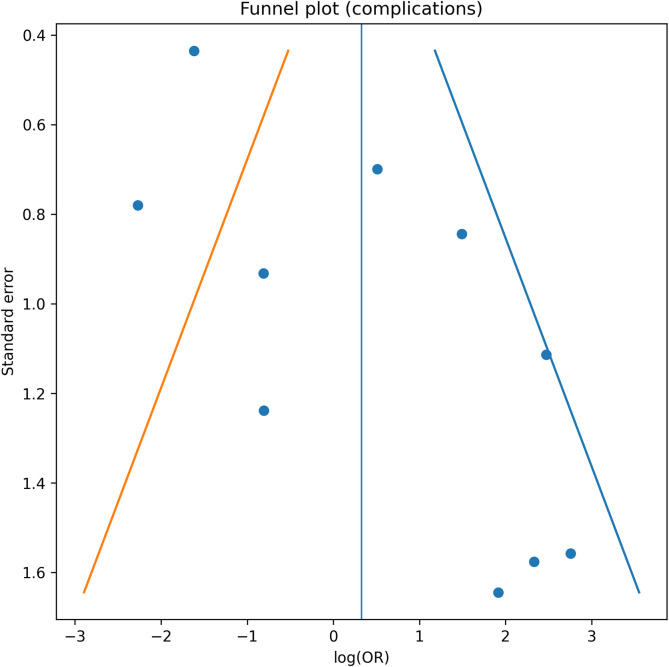
Funnel plot – Complications. Funnel plot assessing publication bias for overall complication outcomes.

Funnel plot inspection suggested mild asymmetry for redisplacement and secondary intervention. Egger’s regression indicated possible small-study effects (Figures 5–8). Restriction to randomized trials preserved both direction and magnitude of effect: redisplacement rates ranged from 24–45% after casting versus 0–8% after K-wire fixation [7–9], confirming robustness of pooled findings.

## Discussion

This systematic review synthesizes contemporary evidence on pediatric distal radius fracture management and supports a biologically informed, stability-based treatment paradigm rather than routine surgical escalation [1, 2, 10]. Across study designs and patient populations, long-term functional recovery was consistently excellent. The principal clinical challenge is not fracture healing, but prevention of early mechanical failure in unstable patterns [2, 3, 15].

### Mechanical stability versus functional outcome

Adjunctive percutaneous K-wire fixation significantly reduces redisplacement and secondary intervention in unstable displaced metaphyseal fractures [7, 8, 10]. In cast-treated cohorts, redisplacement occurs in approximately one-quarter to one-third of cases, whereas primary pin fixation reduces this risk to very low levels [7, 8]. The clinical value of fixation therefore lies in preventing early loss of reduction and repeat procedures.

However, this mechanical advantage does not translate into superior long-term function. Randomized and comparative studies demonstrate equivalent union, wrist motion, and activity levels regardless of fixation [4, 7, 8]. Large contemporary series confirm that K-wires do not eliminate all re-angulation but primarily reduce early instability [16]. Radiographic alignment and patient-centered functional outcome are therefore not synonymous in children, reflecting the remodeling capacity of the distal radius [11, 13, 14].

### Immobilization strategy

Immobilization intensity should align with fracture stability. For stable injuries, particularly torus fractures and minimally angulated metaphyseal fractures, removable immobilization is equivalent to casting in functional recovery and fracture stability [23, 24]. These findings are consistent with contemporary guideline recommendations discouraging rigid casting for buckle fractures [27].

Cast length (above-elbow versus below-elbow) does not improve maintenance of reduction when molding is adequate [21, 22]. Stability depends more on fracture characteristics and cast quality than elbow immobilization. For displaced fractures after reduction, sugar-tong–based splinting demonstrates comparable alignment outcomes and low re-intervention rates when applied within structured protocols [25, 26]. These data support graduated immobilization based on fracture stability rather than uniform casting.

### Casting quality

Casting failure reflects interaction between intrinsic fracture instability and technique-related factors [28]. Complete displacement, fracture obliquity, and associated ulnar fracture are dominant instability drivers [29]. Casting indices, including the cast index and three-point index, correlate with redisplacement in selected pediatric metaphyseal fractures and function best as adjunctive quality markers rather than standalone thresholds [28, 29]. Their predictive value varies with fracture phenotype and reduction quality. Optimal molding improves stability but does not overcome fundamentally unstable fracture patterns.

### Identifying fractures that benefit from fixation

Selective fixation is justified in fractures at high risk of early mechanical failure: complete displacement, translation >50%, associated distal ulna fracture, difficulty maintaining reduction, and limited remaining growth [2, 3, 15]. Age and skeletal maturity are critical modifiers. Remodeling capacity diminishes with advancing maturity, and acceptable deformity thresholds narrow in older children and adolescents [30]. Randomized comparative evidence confirms that unstable fractures in this group frequently fail casting and benefit from primary stabilization [7, 8].

Conversely, evidence supports reconsideration of routine reduction in young children with substantial remaining growth. Completely displaced metaphyseal fractures in children younger than approximately 10–11 years demonstrate reliable union and predictable remodeling even when bayonet apposition is accepted [11]. Early anatomic perfection is therefore not universally required. Chronologic age alone is an imperfect surrogate for remodeling potential. Skeletal maturity occurs earlier in girls than in boys, and treatment decisions should consider remaining growth rather than rigid age cutoffs [30].

### Metaphyseal versus physeal injuries

Metaphyseal and physeal distal radius fractures are biologically distinct and carry different clinical priorities. In metaphyseal fractures, the dominant concern is early mechanical failure and redisplacement. In contrast, physeal injuries are defined by the risk of growth disturbance and premature arrest rather than redisplacement alone [5, 31]. Transphyseal fixation introduces a potential iatrogenic threat to the growth plate and must be judiciously weighed against instability [20]. Accordingly, management of physeal injuries prioritizes restoration of physeal alignment and structured longitudinal surveillance to ensure symmetric growth [5, 31]. Displacement metrics also differ by subtype: metaphyseal fractures are characterized by angulation and translation, whereas physeal displacement is more appropriately assessed by physeal alignment and metaphyseal fragment gap or step-off. Treatment algorithms must therefore distinguish these entities rather than apply uniform radiographic thresholds.

### Complications and surgical context

Overall complication rates were low across treatment strategies [7, 8]. Cast-related complications primarily reflected mechanical failure, whereas K-wire complications were typically minor and self-limited [6, 16]. More invasive alternatives – including elastic intramedullary nailing and plating – carry greater implant-related morbidity and should be reserved for specific indications such as irreducible, intra-articular, or near-mature fractures [10, 30].

### Proposed framework

**Figure 9:**
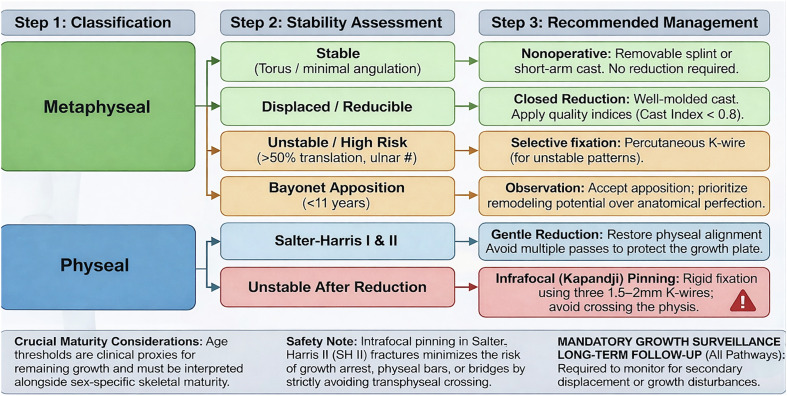
Evidence-based treatment framework for pediatric distal radius fractures. The algorithm categorizes management based on fracture biology (metaphyseal vs. physeal) and mechanical stability. Metaphyseal Pathway: Focuses on graduated immobilization. Stable injuries (e.g., Torus) are managed with minimal intervention. Displaced but stable injuries receive closed reduction and molded casting. Fixation is selectively reserved for “High Risk” patterns to prevent early redisplacement. In children under 11 years, bayonet apposition is often acceptable due to high remodeling potential. Physeal Pathway: Prioritizes growth plate preservation. For unstable SH II injuries, intrafocal (Kapandji) K-wire pinning provides rigid fixation while strictly avoiding transphyseal iatrogenic injury. *Note*: Chronological age thresholds are clinical proxies; treatment decisions must be adjusted based on individual growth velocity and sex-specific maturity.

The proposed algorithm integrates fracture stability, skeletal maturity, and fracture subtype. Where high-quality comparative evidence exists, recommendations are direct; elsewhere, guidance reflects synthesis of best available data and established principles of pediatric fracture biology (Figure 9).

### Limitations

Clinical heterogeneity in fracture definitions, immobilization techniques, and redisplacement thresholds limits precision of pooled estimates. Observational comparisons introduce potential confounding by indication, although consistency of effect across randomized trials mitigates this concern [7, 8]. Functional outcomes were inconsistently reported, and cost-effectiveness data remain limited. Funnel plot asymmetry suggests possible small-study effects; however, effect direction remained stable in sensitivity analyses [10].

## Conclusions

Pediatric distal radius fractures demonstrate predictable healing and excellent long-term function when treatment is aligned with fracture biology and skeletal maturity rather than radiographic perfection alone. Metaphyseal and physeal injuries require distinct consideration: metaphyseal fractures are primarily threatened by early mechanical instability, whereas physeal injuries demand vigilance for growth disturbance and symmetric remodeling.

Nonoperative management remains appropriate for the majority of fractures, including acceptance of bayonet apposition in children with substantial remaining growth. Removable splints, short-arm casting, and graduated immobilization strategies are safe and effective for stable patterns. Percutaneous K-wire fixation should be reserved for fractures at high risk of early failure – particularly unstable displaced patterns in older children and adolescents with diminishing remodeling capacity – where its principal benefit lies in preventing redisplacement and secondary intervention, not in improving ultimate functional outcome.

The Ccentral message of this review is clear: stability and skeletal maturity – not uniform radiographic thresholds – should guide management. A selective, maturity- and stability-based strategy minimizes unnecessary intervention while preserving the excellent natural history of pediatric distal radius fractures.

## Data Availability

All data generated or analyzed during this study are included in this published article.

## Supplementary material

*Table S1*: Randomized control trials and other evidence base studies. *Table S2*: Technique-focused series and noncomparative cohorts included in qualitative synthesis (not pooled in meta-analysis). The supplementary material of this article is available at https://www.sicot-j.org/10.1051/sicotj/2026032/olm.
