# Supplementary material for: Management of pediatric distal radius fractures – A systematic review and meta-analysis
Source: SICOT J. 2026 Jun 5;12:36. doi: 10.1051/sicotj/2026032 (PMC13241055; doi:10.1051/sicotj/2026032)
Supplement: Supplementary file 1 — Table S1: Randomized control trials and other evidence base studies. [file sicotj-12-36-s1.pdf]

Table S1. Randomized control trials and other evidence base studies

Study (design)	Population	Fracture type	Indication	Immobilization compared	Key outcomes (extractable data)
RCT, distal third forearm fractures	Children (n=102)	Distal third forearm (mixed radius/ulna patterns)	Post-reduction casting	Above-elbow vs below-elbow cast	Met criteria for remanipulation: 42% (23/55) vs 31% (14/45); only 4 underwent remanipulation; complications similar
RCT, displaced distal third forearm fractures	Children ≥4 years (n=113 analyzed)	Distal third forearm	Post-reduction casting	Long-arm vs short-arm plaster cast	No significant difference in radiographic maintenance; lost reduction linked to higher cast index; short-arm missed fewer school days / less ADL assistance
RCT, minimally angulated distal radius fractures	5–12 years (n=96)	Metaphyseal distal radius	Stable/minimally angulated (no reduction)	Prefabricated wrist splint vs short-arm cast	6 progressed to 25° at 4 weeks (3 vs 3), none needed surgery; minor device-related issues differ; splint preferred by many families
Equivalence RCT, torus fractures	4–15 years (n=965)	Distal radius torus	Stable	Offer bandage + discharge vs "rigid immobilization"	Pain equivalence at day 3; rigid arm 95% removable wrist splint; complications 1.0% vs 0.6%; school absence OR 0.79; measurable escalation/crossover behavior
RCT, displaced fractures after reduction	4–12 years (n=71)	Distal radius ± distal both-bone	Post-reduction	Double sugar-tong splint vs long-arm cast	Loss ≥10°: 7 vs 2; met remanipulation criteria: 10 vs 5; DSTS overwrapped after week 1 and later converted to short-arm casting
Series (protocol), displaced fractures after reduction	2–12 years (n=53)	Mostly metaphyseal; small physeal subset	Post-reduction	Sugar-tong → short-arm cast at 2–3 weeks	Maintained reduction 96% (51/53); swelling follow-up within 1 week; planned conversion at ~3 weeks if tenderness low and alignment maintained
