# Supplementary material for: Management of pediatric distal radius fractures – A systematic review and meta-analysis
Source: SICOT J. 2026 Jun 5;12:36. doi: 10.1051/sicotj/2026032 (PMC13241055; doi:10.1051/sicotj/2026032)
Supplement: Supplementary file 2 — Table S2: Technique-focused series and noncomparative cohorts included in qualitative synthesis (not pooled in meta-analysis). [file sicotj-12-36-s2.pdf]

Supplementary Table S2. Technique-focused series and noncomparative cohorts included in qualitative synthesis (not pooled in meta-analysis)

Study	Design	N	Population / Fracture type	Intervention	Follow-up	Key outcomes (high-yield)	Why included
Nietosvaara 2005 [13]	Observational cohort	109	Distal radial physeal fractures	Closed reduction + cast (noncomparative)	Immobilization 5.7 wks	48% healed in malunion; marked initial displacement predicted complications/redisplacement; emphasizes risk stratification and “casting failure” biology	Key evidence for instability predictors and limits of casting in physeal injuries
Ramoutar 2015 [14]	Retrospective series	248	Extra-articular distal radius fractures (closed), pediatric	Closed reduction + K-wire fixation	Mean follow-up 6.6 wks (range 4–156)	17% K-wire complications; 6.9% required additional GA (revision or deep migrated wire removal); 87% normal function; K-wires do not fully prevent re-angulation	Large real-world series clarifying benefits and limitations of K-wires
Satish 2014 [15]	Case series	46 (completed follow-up)	Completely displaced pediatric distal radius fractures	Kapandji leverage intrafocal + extrafocal K-wires + cast	Short-term (minimum several months)	No loss of reduction, no pin-related complications, all healed with full function; mean K-wiring time 7 min	Technique-focused evidence supporting intrafocal leverage efficacy in high displacement
Valisena 2019 [16]	Retrospective series	56	Unstable displaced metaphyseal + SH-II	Kapandji intrafocal technique, standardized stability test	Mean follow-up 18 months	No pin-related complications; full function in all; rare epiphysiodesis described in SH-II context	Adds longer follow-up for Kapandji technique and physeal monitoring

Study	Design	N	Population / Fracture type	Intervention	Follow-up	Key outcomes (high-yield)	Why included
Bassi 2024 [17]	Retrospective cohort (single arm)	195	Pediatric displaced distal radius fractures	Modified Kapandji technique	Follow-up reported in paper	Reduction “good to anatomic” in 85%; overall complications 15% (serious ≈1%); metaphyseal fractures showed greater angulation change; informs fixation strategy by fracture subtype	Contemporary “large series” informing technique refinement and complication expectations
Jerome 2021 [18]	Retrospective series	20	Salter-Harris II distal radius	Intrafocal K-wire technique	Mean 4 years	Excellent radiographic/functional outcomes; no growth arrest reported in series; supports physeal-respecting approach	Supports intrafocal technique as selective option in SH-II
